# 

**Published:** 1881-01

**Authors:** 


### CONTENTS.

| | Page. | | Page. |
|---|---|---|---|
| Dyspepsia | 3 | The Gulf Stream | 20 |
| Whooping Cough | 6 | The Mouth as a Pocket | 21 |
| Old Young People and Young Old People | 7 | The Drunkard (poetry) | 22 |
| Cancer | 9 | Tom Hunt | 23 |
| Burying Alive | 10 | Atmospheric Dust | 28 |
| Accidents and Emergencies | 13 | Iron Smelting | 29 |
| Oxygen | 17 | Beautiful Snow (poetry) | 30 |
| Praise Your Wife | 18 | Western Texas as a Health Resort | 31 |
| | | Creosote for Bronchitis and Catarrh | 34 |

